# Cystathionine β-synthase (CBS) deficiency suppresses erythropoiesis by disrupting expression of heme biosynthetic enzymes and transporter

**DOI:** 10.1038/s41419-019-1951-0

**Published:** 2019-09-24

**Authors:** Peng Zhao, Christopher Qian, Yun-Jin Chen, Yuan Sheng, Ya Ke, Zhong-Ming Qian

**Affiliations:** 1 Laboratory of Neuropharmacology, Fudan University School of Pharmacy, Shanghai, 201203 China; 2 National Clinical Research Center for Aging and Medicine, Huashan Hospital, FudanUniversity, Shanghai, 200040 China; 3 School of Biomedical Sciences and Gerald Choa Neuroscience Centre, Faculty of Medicine, The Chinese University of Hong Kong, Shatin, NT Hong Kong; 4 Institute of Translational & Precision Medicine, Nantong University, Nantong, JS 226019 China

**Keywords:** Homeostasis, Anaemia

## Abstract

The reduced iron usage induced by the suppression of erythropoiesis is a major cause of the systemic iron overload in CBS knockout (CBS^−/−^) mice. However, the relevant mechanisms are unknown. Here, we examined changes in granulocyte/erythroid cell ratios, iron content, and expression of iron-metabolism proteins, including; two key enzymes involved in the heme biosynthetic pathway, ALAS2 (delta-aminolevulinate synthase 2) and FECH (ferrochelatase), a heme exporter from the cytosol and mitochondria, FLVCR (feline leukemia virus subgroup C cellular receptor) as well as EPO (erythropoietin), EPOR (erythropoietin receptor) and HIF-2α (hypoxia inducible factor-2 subunit α), in the blood, bone marrow or liver of CBS^−/−^ (homozygous), CBS^+/−^ (heterozygous) and CBS^+/+^ (Wild Type) mice. Our findings demonstrate that CBS deficiency can induce a significant reduction in the expression of ALAS2, FECH, FLVCR, HIF-2α, EPO, and EPOR as well as an increase in interleukin-6 (IL-6), hepcidin and iron content in the blood, bone marrow or liver of mice. We conclude that the suppression of erythropoiesis is mainly due to the CBS deficiency-induced disruption in the expression of heme biosynthetic enzymes and heme-transporter.

In addition to CSE (cystathionine gammalyase)^1^ and MST (3-mercaptopyruvate sulfurtransferase)^2,3^, CBS (Cystathionine β- synthase) is a key enzyme for hydrogen sulfide (H2S) production from L-cysteine^4,5^. CBS^−/−^ (homozygous knockout) mice rarely survive past 4 weeks of age^4^, implying that CBS is essential for survival and development in mice. In a recent study, we found that CBS knockout (CBS^−/−^) mice exhibited anemia and a significant increase in iron content in the serum, liver, spleen, and heart, along with severe damage to the liver, displaying a hemochromatosis-like phenotype^6^. Also, we demonstrated that this iron-related phenotype could partially be reversed by administration of CBS-overexpressing adenovirus. These findings showed that CBS is required for body iron homeostasis^6^.

The reduced iron usage due to suppressed erythropoiesis is a major cause of the systemic iron overload^6^. However, the molecular mechanisms by which CBS deficiency suppresses erythropoiesis are unknown. In this study, we first examined in detail the effects of CBS^−/−^ on red blood cell (RBC), hematocrit (HCT), hemoglobin (Hb), mean corpuscular hemoglobin (MCH), mean corpuscular volume (MCV), iron, interleukin-6 (IL-6), and hepcidin contents in the blood, and the ratio of granulocyte/erythroid cells, iron, ferritin-light chain (Ft-L) and ferritin-heavy chain (Ft-H) levels, as well as delta-aminolevulinate synthase 2 (ALAS2) and ferrochelatase (FECH), two key enzymes involved in the heme biosynthetic pathway, and feline leukemia virus subgroup C cellular receptor (FLVCR), a heme exporter from the cytosol and mitochondria in the bone marrow of CBS^−/−^ (homozygous), CBS^+/−^ (heterozygous), and CBS^+/+^ (Wild Type) mice.

We then investigated the expression of iron transport proteins, including transferrin receptor 1 (TfR1), divalent metal transporter 1 (DMT1), ferroportin 1 (Fpn1), iron storage proteins Ft-L and Ft-H, iron regulatory hormone (hepcidin) and proteins (IRPs), and other relevant molecules, including erythropoietin (EPO) and its receptor (EPOR), hypoxia inducible factor-2 subunit α (HIF-2α), phosphorylated Janus kinase 2 (p-JAK2) and erythroferrone (ERFE) in the bone marrow, bone marrow-derived macrophages (BMDMs) or liver of CBS^−/−^, CBS^+/−^, and CBS^+/+^ mice.

Our findings demonstrated that CBS deficiency could induce a significant reduction in the expression of ALAS2, FECH, and FLVCR, alongside a significant increase in iron content in the bone marrow, indicating that the suppression of erythropoiesis is mainly due to the inhibitory effects of CBS deficiency on two key enzymes involved in the heme biosynthetic pathway, and also on the transport of heme from the cytosol and mitochondria. The reduced expression of EPO in CBS^−/−^ mice could lead to an increase in CFU-E (colony-forming unit erythroids) apoptosis and hence reduce the number of erythroblasts, which may also be one of the molecular mechanisms involved in the suppression of erythropoiesis in CBS^−/−^ mice.

## Results

### CBS deficiency induced a reduction in RBC, Hb, and HCT, and an increase in MCH, MCV, iron, IL-6, and hepcidin in the blood

**Fig. 1:**
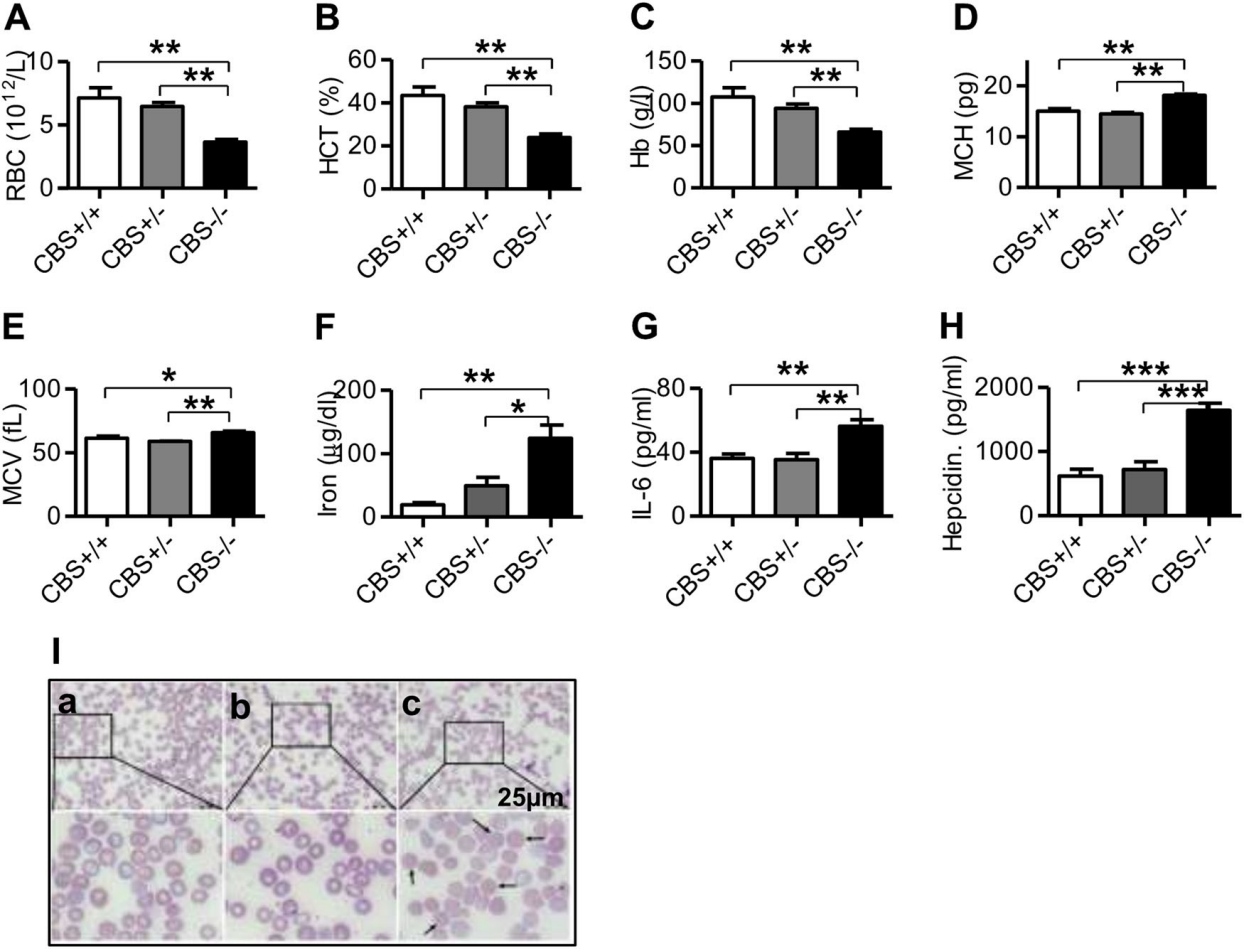
CBS deficiency induced a reduction in RBC, Hb, and HCT, and an increase in MCH, MCV, iron, IL-6, and hepcidin in the blood. RBC (**a**), Hb (**b**), HCT (**c**), MCH (**d**), MCV (**e**), iron (**f**), IL-6 (**g**) and hepcidin (**h**) in the blood and blood smear (I, ×1000) of CBS^+/+^ (**a**), CBS^+/−^ (**b**) and CBS^−/−^ (**c**) mice were measured as described in the “Materials and Methods” section. Data are means ± SEM (*n* = 6). *p < 0.05, **p < 0.01, ***p < 0.001 vs. CBS^+/+^ or CBS^+/−^ mice

To find out the effects of CBS deficiency on erythropoiesis, we initially measured RBC, Hb, HCT, MCH, MCV and also the contents of iron, IL-6 and hepcidin in the blood of CBS^+/+^, CBS^+/−^, and CBS^−/−^ mice. The contents of RBC (Fig. 1a), Hb (Fig. 1b), and HCT (Fig. 1c) were found to be significantly lower, while the levels of MCH (Fig. 1d) and MCV (Fig. 1e) higher in CBS^−/−^ mice when compared to CBS^+/+^ and CBS^+/−^ mice. There were no statistically significant differences in these measurements between CBS^+/+^ and CBS^+/−^ mice. The findings were consistent with the changes in erythrocyte morphologic characteristics (Fig. 1i). Observation of blood smear showed that the erythrocytes were uniform in size in CBS^+/+^ mice (Fig. 1Ia), whereas the central pale area of erythrocytes was enlarged and annular erythrocytes were visible in CBS^+/−^ mice (Fig. 1Ib), and in CBS^−/−^ mice, the central pale area of erythrocytes was reduced or largely disappeared and spherical erythrocytes were visible (Fig. 1Ic). These findings demonstrated that CBS deficiency could induce a significant reduction in RBC, Hb, HCT, an increase in MCH and MCV, and the formation of spherical erythrocytes. In addition, the contents of iron (Fig. 1f), IL-6 (Fig. 1g) and hepcidin (Fig. 1h) in the blood of CBS^−/−^ mice were significantly higher than those in the CBS^+/+^ and CBS^+/−^ mice.

### CBS deficiency increased the ratio of granulocyte/erythroid cells, iron, Ft-L and Ft-H contents, and reduced the expression of ALAS2, FECH, and FLVCR mRNAs in the bone marrow

**Fig. 2:**
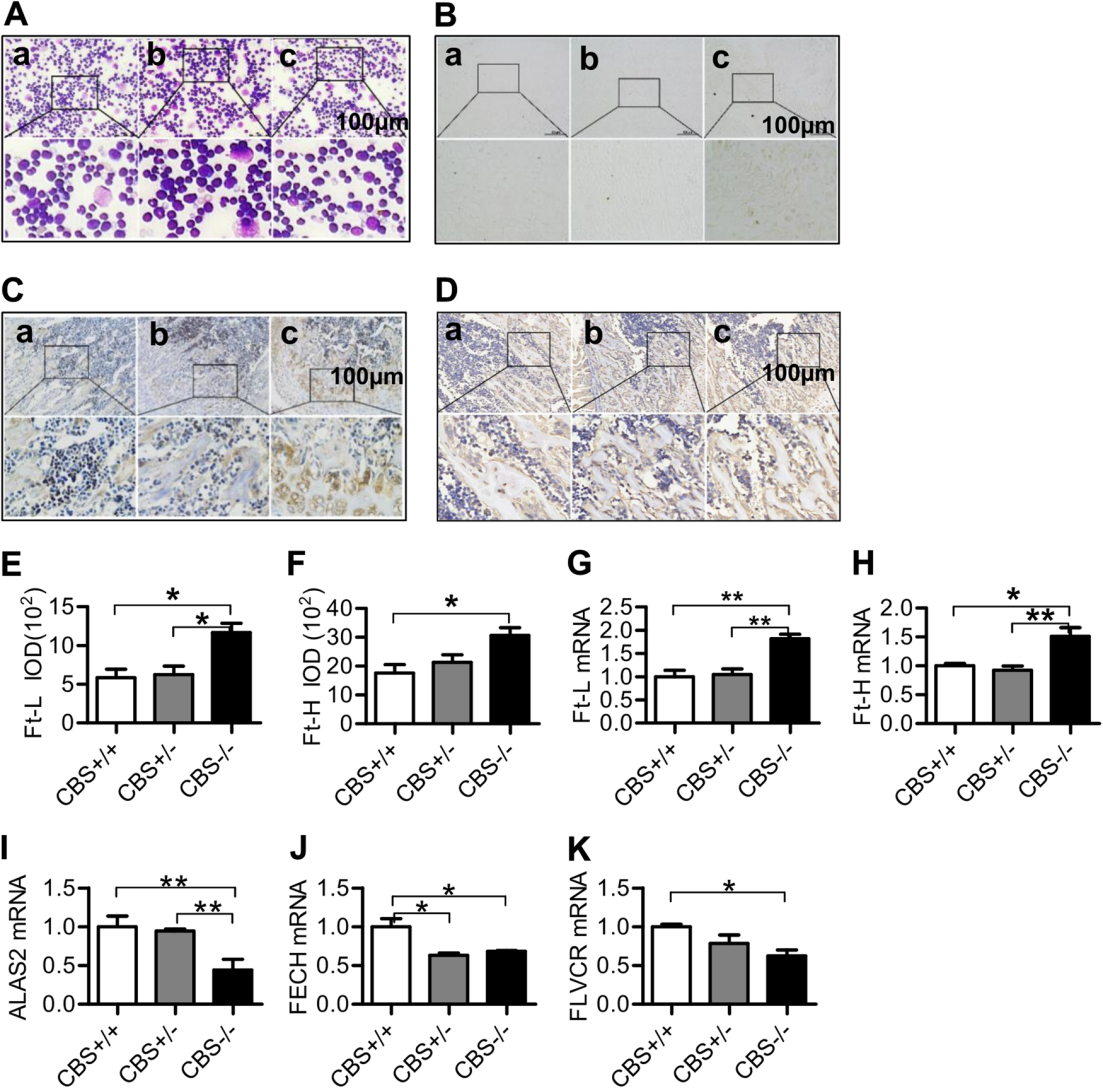
CBS deficiency increased the ratio of granulocyte/erythroid cells, iron, Ft-L, and Ft-H contents, and reduced expression of ALAS2, FECH, and FLVCR mRNAs in the bone marrow. **a** Bone marrow smear (granulocyte/erythroid cells); **b** Prussian blue staining (iron) in the bone marrow; **c**–**j** Immunofluorescence analysis of the expression of Ft-L (**c** and **e**) and Ft-H (**d** and **f**) proteins in the bone marrow; and G-K: Real-time PCR analysis of the expression of Ft-L (**g**), Ft-H (**h**), ALAS2 (**i**), FECH (**j**) and FLVCR (K) mRNAs in the bone marrow of CBS^+/+^ (**a**), CBS^+/−^ (**b**), and CBS^−/−^ (**c**) mice. Data are means ± SEM (*n* = 6). *p < 0.05, **p < 0.01 vs. CBS^+/+^ or CBS^+/−^ mice

We then examined the changes occurring in the bone marrow induced by CBS deficiency, and found that there was no abnormal cell morphology in bone marrow smear of CBS^+/+^ mice (Fig. 2Aa). There was no significant difference in the observed bone marrow smear between the CBS^+/+^ mice (Fig. 2Aa) and CBS^+/−^ mice (Fig. 2Ab). However, the ratio of granulocyte/erythroid cells significantly increased and the number of erythroid cells at each stage was reduced in CBS^−/−^ mice when compared with CBS^+/+^ mice (Fig. 2Ac, Supplementary Table 2 and 3).

As Iron is a hematopoietic raw material^7^, we therefore investigated the effect of CBS deficiency on iron content in the bone marrow. Prussian blue staining demonstrated that iron levels in the bone marrow were significantly higher in CBS^−/−^ mice (Fig. 2Bc) than in CBS^+/−^ (Fig. 2Bb) and CBS^+/+^ mice (Fig. 2Ba). Also, the number of sideroblasts was significantly increased in the bone marrow of CBS^−/−^ mice as compared with CBS^+/−^ and CBS^+/+^ mice (Supplementary Table 4 and 5), and the percentage of type IV sideroblasts was remarkably higher in CBS^−/−^ mice (Supplementary Fig. 1c) than in CBS ^+/−^ (Supplementary Fig. 1b) and CBS^+/+^ mice (Supplementary Fig. 1a), demonstrating that CBS deficiency could induce a significant increase in the number of sideroblasts and iron content in erythroblasts of bone marrow (Supplementary Table 4 and 5, and Supplementary Fig. 1). Western blot and RT-PCR analysis revealed a significant increase in Ft-L and Ft-H Proteins (Ft-L: Fig. 2c, e; Ft-H: Fig. 2d, f) and mRNAs (Ft-L: Fig. 2g; Ft-H: Fig. 2h) in the bone marrow of CBS^−/−^ mice, as compared with CBS^+/+^ and CBS^+/−^ mice.

ALAS2 and FECH are the first (rate-limiting) and the last enzymes in the heme biosynthetic pathway, and FLVCR is a heme exporter from the cytosol and mitochondria. We therefore decided to investigate the effect of CBS deficiency on the expression of these molecules in the bone marrow. We did not find any differences in the expression of ALAS2 mRNA between CBS^+/+^ and CBS^+/−^ mice; however, expression of ALAS2 mRNA was shown to be significantly lower in CBS^−/−^ mice than in CBS^+/+^ and CBS^+/−^ mice (Fig. 2i). The expression of FECH mRNA was significantly lower in CBS^−/−^ and CBS^+/−^ mice than in CBS^+/+^ mice (Fig. 2j). A significant reduction was also found in the expression of FLVCR mRNA in the bone marrow of CBS^−/−^ mice, when compared with CBS^+/−^ mice.

### CBS deficiency reduced the expression of TfR1, DMT1, Fpn1, and p-JAK2 proteins and mRNAs, and EPO, EPOR, and ERFE mRNAs in the bone marrow

**Fig. 3:**
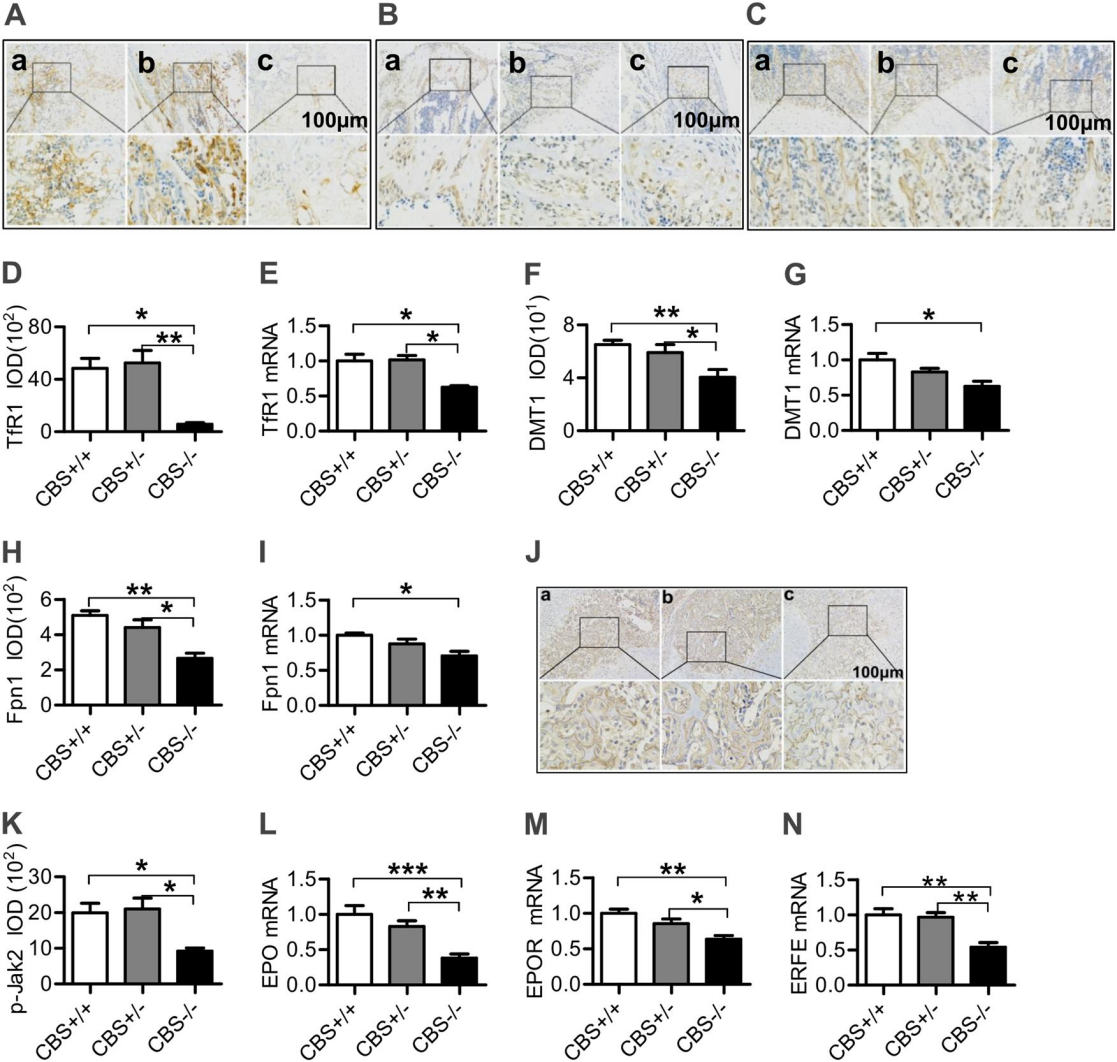
CBS deficiency reduced expression of TfR1, DMT1, Fpn1 and p-JAK2 Proteins and mRNAs and EPO, EPOR, and ERFE mRNAs in the bone marrow. The expression of TfR1 (**a** and **d**), DMT1 (**b** and **f**), Fpn1 (**c** and **h**) and pJAK2 (**j** and **k**) proteins and TfR1 (**e**), DMT1 (**g**), Fpn1 (**i**), EPO (**l**), EPOR (**m**), ERFE (N) mRNAs in the bone marrow of CBS^+/+^ (**a**), CBS^+/−^ (**b**) and CBS^−/−^ (**c**) mice was detected by immunohistochemistry staining and Real-time PCR as described in the “Materials and Methods” section. Data are means ± SEM (*n* = 6). *p < 0.05, **p < 0.01, ***p < 0.001 vs. CBS^+/−^ or CBS^+/+^ mice

We also examined the expression of iron uptake proteins TfR1 and DMT1, and iron release protein Fpn1 in the bone marrow. Immunofluorescence and real-time PCR analysis showed that the expression of TfR1 (Fig. 3a, d, and e), DMT1 (Fig. 3b, f, and g) and Fpn1 (Fig. 2c, h, and i) proteins and mRNAs in the bone marrow was significantly lower in CBS^−/−^ mice than in CBS^+/+^ and/or CBS^+/−^ mice. The expression of proteins involved in cell-iron uptake and release is affected by p-JAK2 (IL-6/p-JAK2/p-STAT3 pathway), EPO/EPOR and ERFE, and we therefore examined the effects of CBS deficiency on the expression of these molecules. Immunohistochemistry staining and real-time PCR analysis showed that the levels of p-JAK2 protein (Fig. 3j, k), EPO (Fig. 3l), EPOR (Fig. 3m), and ERFE (Fig. 3n) mRNAs in bone marrow in CBS^−/−^ mice were significantly lower than those in CBS^+/+^ and CBS^+/−^ mice, demonstrating that CBS deficiency could down-regulate the expression of p-JAK2, EPO, EPOR, and ERFE in bone marrow.

### CBS deficiency up-regulated the expression of TfR1, Ft-L and Ft-H proteins and down-regulated Fpn1 and DMT1 in BMDMs

**Fig. 4:**
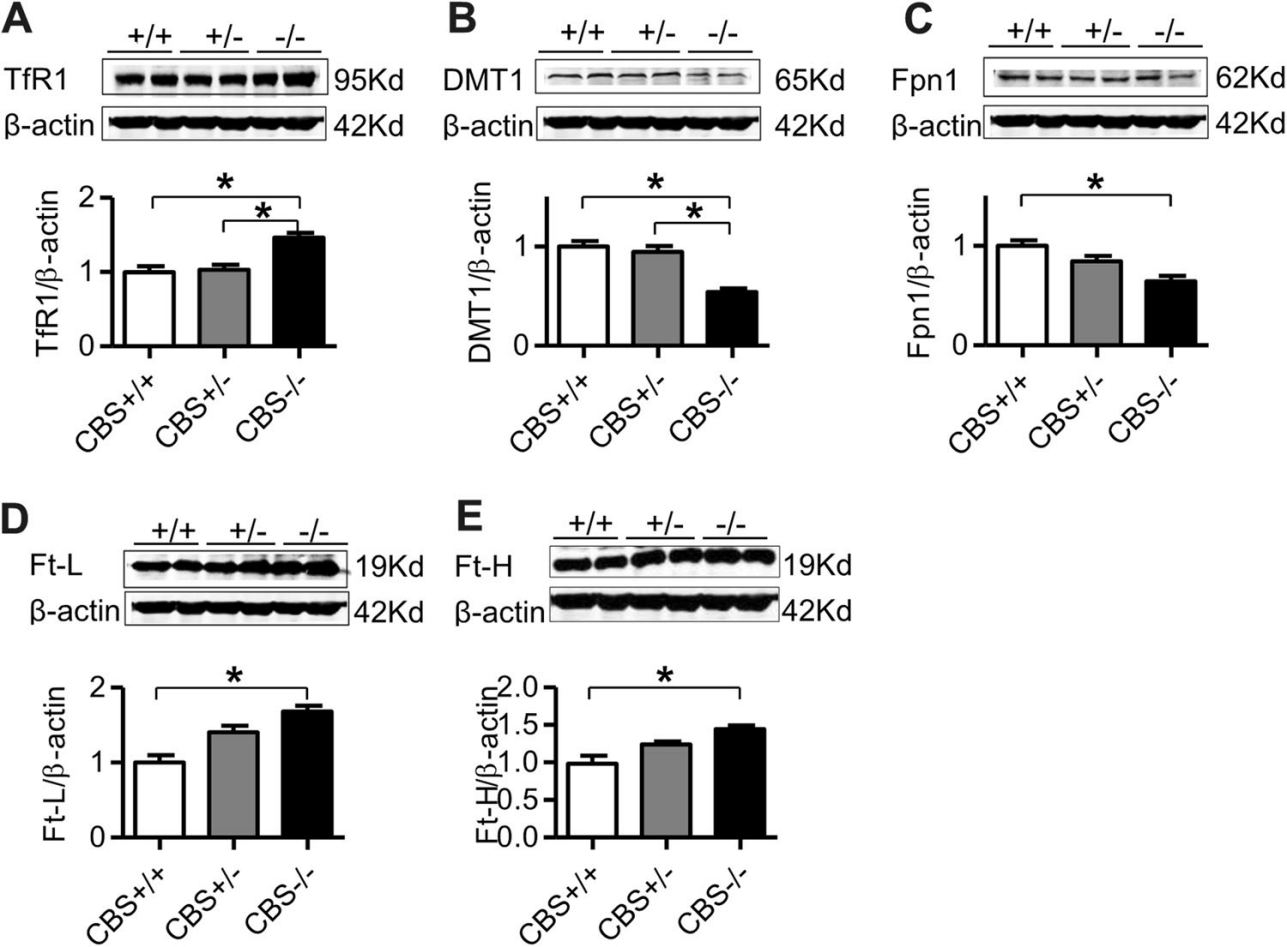
CBS deficiency up-regulated TfR1 and Fts and down-regulated Fpn1 and DMT1 expression in BMDMs. The expression of TfR1 (**a**), DMT1 (**b**), Fpn1 (**c**), Ft-L (**d**) and Ft-H (**e**) in the BMDMs of CBS^+/+^, CBS^+/−^, and CBS^−/−^ mice was measured by Western blot analysis as described in the “Materials and Methods” section. Data are means ± SEM (*n* = 6). *p < 0.05, **p < 0.01, ***p < 0.001 vs. CBS^+/−^ or CBS^+/+^ mice

Because the macrophage plays key functions in heme synthesis^8,9^, we also assessed the changes in the expression of TfR1, DMT1, Fpn1, Ft-L, and Ft-H in the BMDMs of CBS^+/+^, CBS^+/−^, and CBS^−/−^ mice. Western blot analysis demonstrated that the expression of TfR1 (Fig. 4a), Ft-L (Fig. 4d), and Ft-H (Fig. 4e) proteins was significantly higher, while DMT1 (Fig. 4b) and Fpn1 (Fig. 4c) proteins lower in BMDM cells in CBS^−/−^ mice, when compared to CBS^+/+^ and CBS^+/−^ mice.

### CBS deficiency down-regulated expression of HIF-2α, EPO, and IRPs in the liver

**Fig. 5:**
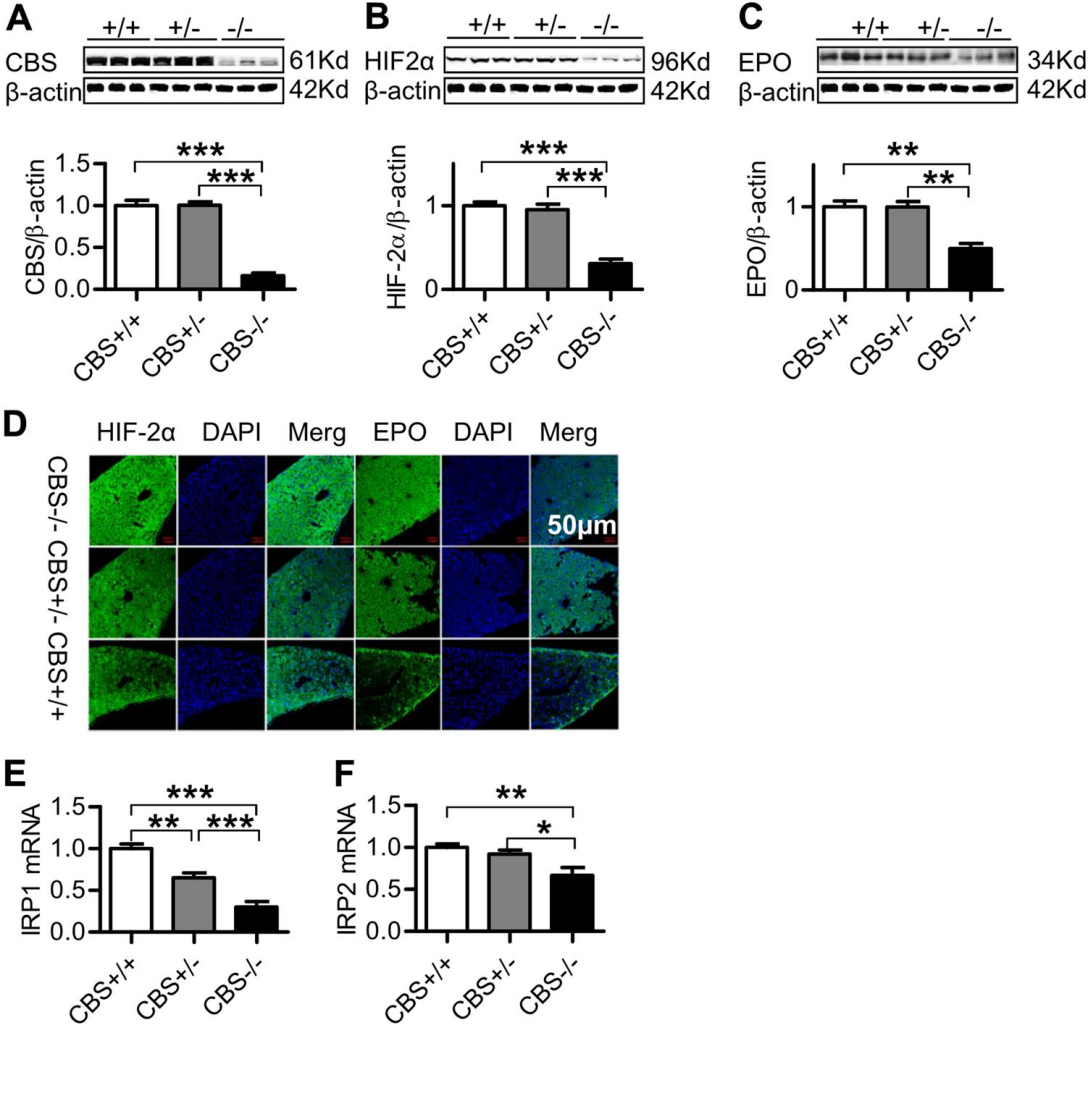
CBS deficiency down-regulated expression of HIF-2α, EPO and IRPs in the liver. The expression of CBS (**a**), HIF-2α (**b**, **d**), EPO (**c**, **d**) proteins in the liver of CBS^+/+^, CBS^+/−^, and CBS^−/−^ mice was examined by Western blot and immunofluorescence (×400) analysis and IRP 1 (**e**) & IRP2 (**f**) mRNAs by Real-time PCR analysis as described in the “Materials and Methods” section. Data are means ± SEM (*n* = 6). *p < 0.05, **p < 0.01, ***p < 0.001 vs. CBS^+/−^ or CBS^+/+^ mice

To further elucidate the mechanisms involved in the suppression of erythropoiesis induced by CBS deficiency, we also investigated the expression of EPO, the upstream regulator HIF-2α and IRPs in the liver. Western blot analysis showed that, in addition to the significantly reduced CBS expression (Fig. 5a), CBS deficiency induced a significant reduction in the expression of HIF-2α (Fig. 5b) and EPO (Fig. 5c) in the liver, being significantly lower in CBS^−/−^ mice when compared to CBS^+/+^ and CBS^+/−^ mice. There were no significant differences in the expression of HIF-2α and EPO between CBS^+/+^ and CBS^+/−^ mice. These findings were also confirmed by immunofluorescence observation (Fig. 5d). In addition, real-time PCR analysis demonstrated that the expression of IRP1 (Fig. 5e) and IRP 2 (Fig. 5f) mRNAs in the liver of CBS^−/−^ mice was significantly lower than that in CBS^+/+^ and CBS^+/−^ mice, implying that CBS deficiency could also down-regulate the expression of IRP1 and IRP2 in the liver.

## Discussion

In this study, we demonstrated that the absence of CBS (CBS^−/−^) can induce a significant reduction in red blood cell, hemoglobin, and hematocrit in peripheral blood, as well as increase the ratio of granulocyte/erythroid cells with reduced numbers of erythroid cells at all stages in the bone marrow of mice. These findings provide solid evidence for CBS^−/−^ having a role in suppressing hemoglobin synthesis and erythropoiesis, indicating that CBS is essential for these physiological activities.

Erythropoiesis (erythroid production process) is a tightly regulated and multi-step process, originating in the bone marrow from a multipotent hematopoietic stem cell (HSC) and terminating in a mature, enucleated red blood cell^10–12^. During terminal erythropoiesis, iron is essential for hemoglobin synthesis^7^. Intracellular iron is targeted to the mitochondria for incorporation into a porphyrin ring to form heme and cytosolic iron–sulfur proteins^13^. In mammals, eight enzymes are involved in the heme biosynthetic pathway, located successively in the mitochondria, the cytosol and the mitochondria. ALAS, the first enzyme of the pathway which catalyze the condensation of glycine and succinyl CoA to form delta-aminolevulinic acid (ALA), plays a critical role in controlling the heme biosynthesis^14^. FECH is the last enzyme of the pathway, located in the mitochondrial inner membrane and catalyzes the insertion of Fe(II) iron into protoporphyrin IX (PPIX)^14^. FLVCR is an exporter of heme from the cytosol and mitochondria^15,16^.

**Fig. 6:**
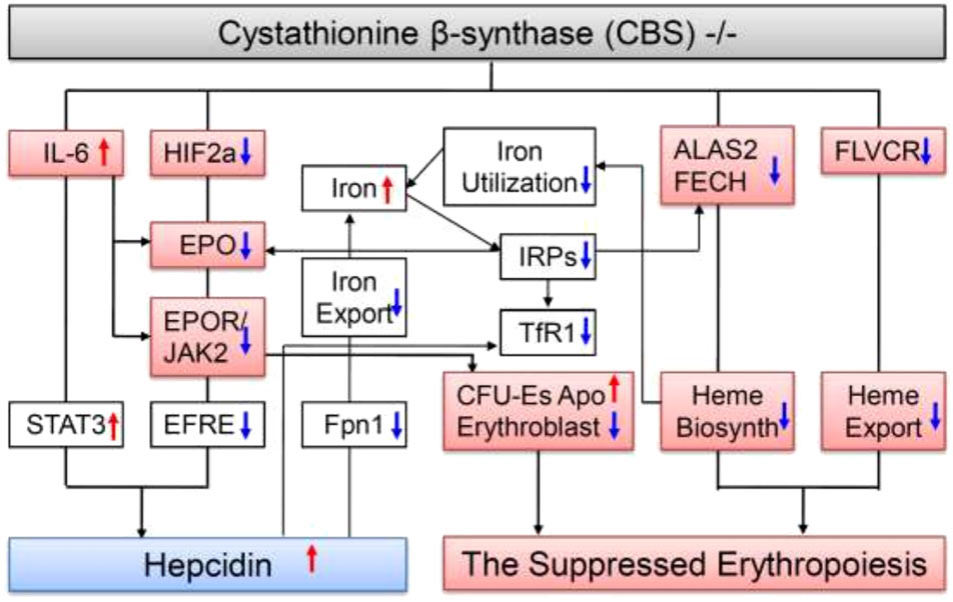
A hypothetical scheme for the mechanisms involved in the suppressed erythropoiesis induced by CBS deficiency. CBS deficiency suppressed erythropoiesis mainly by disrupting expression of heme biosynthetic enzymes and transporter. CBS deficiency could induce a significant reduction in the expression of ALAS2 and FECH, two key enzymes involved in the heme biosynthetic pathway, and FLVCR, an exporter of heme from the cytosol and mitochondria, inhibiting heme biosynthesis and transport of heme from the cytosol and mitochondria in the bone marrow. Also, CBS deficiency could down-regulate HIF-2α and up-regulate IL-6 expression, and then inhibit EPO expression, and the reduced expression in EPO could increase apoptosis of CFU-Es and reduce the number of erythroblasts, which may also contribute to the suppressed erythropoiesis in CBS deficiency mice. In addition to the reduced iron utilization, the increased iron contents in the erythroblasts and bone marrow may be partly associated the reduced Fpn1 expression and then iron release from the bone marrow cells in CBS^−/−^ mice. The up-regulation of hepcidin in CBS^−/−^ mice may be due to the increased expression of IL-6 (IL-6/STAT3 pathway) and reduced contents of HIF2a/EFRE (HIF2a/EPO/EPOR/EFRE pathway). (ALAS2 delta-aminolevulinate synthase, CBS Cystathionine β-synthase, EPO Erythropoietin, EPOR EPO receptor, FECH Ferrochelatase, FLVCR Feline leukemia virus subgroup C cellular receptor, Fpn1 Ferroportin 1, HIF-2α Hypoxia inducible factor-2 subunit α, IL-6 Interleukin-6, IRPs Iron regulatory proteins, p-JAK2 Phospho-Janus Kinase 2, STAT3 Signal transducer and activator of transcription 3, TfR1 Transferrin receptor 1)

In this study, we found that CBS^−/−^ could induce a significant reduction in the expression of ALAS1, FECH, and FLVCR and an increase in the iron content of erythroblasts and bone marrow. These findings imply that CBS deficiency suppresses erythropoiesis, mainly by inhibiting expression of heme biosynthetic enzymes and transporters, disrupting heme biosynthesis and reducing heme export from the cytosol and mitochondria (Fig. 6). The significant increase in iron content of the erythroblasts and bone marrow may mainly be due to the reduction of iron utilization induced by disrupted heme biosynthesis and export, and may also be one of the reasons for the reduced expression of IRP1 and TfR1 in the bone marrow (Fig. 6).

Terminal erythropoiesis occurs in anatomic niches known as erythroblastic islands^12^. Erythroblastic islands are unique to mammalian erythropoiesis and consist of a central macrophage surrounded by up to 30 erythroid cells at varying degrees of red cell maturation^17^. The cells range from colony-forming unit-erythroid cells (CFU-Es) to enucleating erythroblasts, and are the site of hemoglobin synthesis by terminally differentiating erythroblasts^12,18^. It has been well documented that CFU-Es express abundant EPOR^19^. CFU-Es and their progeny divide several times generating 8–64 erythroblasts within 7–8 days in the presence of EPO, whereas CFU-Es undergo apoptosis in the absence of EPO^20^. HIF-2α (also known as EPAS1, endothelial PAS domain protein 1 or HLF, HIF-like factor) is the main regulator of EPO production^21–23^. The expression of HIF-2α has been identified in endothelial cells, hepatocytes, and renal peritubular interstitial cells^24,25^. Renal and hepatic EPO synthesis is predominantly HIF-2α-regulated in response to hypoxia^21,25,26^. The significant reduction in EPO and EPOR/JAK2 found in the bone marrow and liver, and in HIF-2α in the liver of CBS^−/−^ mice suggests that CBS deficiency can inhibit EPO expression by a HIF-2α-mediated process and that the reduced expression in EPO may increase apoptosis of CFU-Es and reduce the number of erythroblasts, which may also contribute to the suppressed erythropoiesis in CBS deficient mice (Fig. 6).

Inflammation has been demonstrated to negatively affect the synthesis and biological activity of EPO^27^, due to the combination of direct inhibition via cytokines^28^ as well as the expression of EPOR on erythroid progenitors^29,30^. It has also been demonstrated that CBS^−/−^ can up-regulate the expression of IL-6 in the liver^6^. In this study, we demonstrated that EPO and EPOR expression in bone marrow (erythroid progenitors) is significantly reduced, while IL-6 in serum is increased in CBS^−/−^ mice. These findings indicate that in addition to HIF-2α, the increased inflammatory cytokine IL-6 may also be associated with the reduced expression of EPO and EPOR, and subsequently ineffective erythropoiesis in CBS^−/−^ mice (Fig. 6).

In addition to the reduced iron utilization, the increased iron content in erythroblasts and bone marrow may be partly associated with the reduced Fpn1 expression and iron release from bone marrow cells in CBS^−/−^ mice. Fpn1 is the only identified cellular iron exporter, and is also a receptor for hepcidin^31^. Hepcidin is a secreted peptide hormone encoded by the HAMP (hepcidin antimicrobial peptide) gene and produced predominantly by the liver^32^. After binding with hepcidin, Fpn1 is internalized and subsequently degraded^31^. The contents of hepcidin were found to be significantly increased in the blood of CBS^−/−^ mice when compared with CBS^+/+^ or CBS^+/−^ mice. The significant increase in hepcidin in CBS^−/−^ mice could lead to a significant reduction in Fpn1 and iron export from cells, which may also contribute to the increased iron content in the erythroblasts and bone marrow (Fig. 6). Also, TfR1 has been found to be directly inhibited by hepcidin in different types of cells^33,34^. Therefore, the increase in hepcidin is highly likely to be one of the causes of the reduced expression of TfR1 in the bone marrow (Fig. 6).

Increased expression of IL-6 will activate the JAK2/STAT2 pathway, leading to an increase in the expression of hepcidin^35^. In addition, the increased expression of hepcidin may also be partly due to the reduced expression of ERFE in CBS^−/−^ mice. ERFE is a glycoprotein with 326-amino-acid residues and has recently been identified as another regulator of iron homeostasis^36^. ERFE is produced by erythrocytic progenitors in response to EPO to increase iron absorption and mobilization of iron from stores by down-regulating hepcidin expression^37^. The reduced expression of EFRE in CBS^−/−^ mice would remove the inhibitory effects of EFRE on hepcidin expression, leading to an increase in the expression of hepcidin (Fig. 6).

In summary, our findings firstly, provide solid evidence to support the idea that CBS is essential for hemoglobin synthesis and erythropoiesis, and also demonstrate for the first time that CBS deficiency suppresses erythropoiesis, mainly by disrupting the expression of ALAS2 and FECH, two key enzymes involved in the heme biosynthetic pathway, as well as FLVCR, an exporter of heme from the cytosol and mitochondria. Secondly, our data show that reduced EPO-induced increase in ‘CFU-Es’ apoptosis and the reduction in the number of erythroblasts may also be one of causes for the suppressed erythropoiesis in CBS^−/−^ mice. Thirdly, our results imply that, in addition to the reduced iron utilization, the increased iron content in erythroblasts and bone marrow may be partly associated with reduced Fpn1 expression and iron release from bone marrow cells in CBS^−/−^ mice. Finally, our study suggests that the up-regulation of hepcidin in CBS^−/−^ mice may be due to the increased expression of IL-6 and reduced HIF2a/EFRE contents (Fig. 6). These findings constitute important contributions to the knowledge of how our body can maintain iron homeostasis and may also be critical in elucidating the causes of patients with suppressed erythropoiesis.

## Materials and methods

### Materials

Unless otherwise stated, all chemicals were obtained from the Sigma Chemical Company, St. Louis, MO, USA. Mouse monoclonal anti-rat TfR1 was purchased from Invitrogen, Carlsbad, CA, USA; rabbit polyclonal anti-Fpn1 from Novus Biologicals, Littleton, CO, USA; mouse anti-p-JAK2 (phospho-Janus Kinase 2) from Cell Signaling Technology, Boston, USA; TRIzol reagent from Life Technologies, Carlsbad, CA, USA; and AevertAid First Strand cDNA Synthesis Kit from Thermo Scientific, Waltham, MA, USA. Rabbit polyclonal anti-Ft-Land anti-DMT1 were purchased from Protein-tech, Chicago, IL, USA; rabbit polyclonal anti-CBS, HIF-2α and EPO antibodies from Abcam, Inc., Cambridge, UK; rabbit polyclonal anti-Ft-H from Bioworld Technology Inc., Louis Park, MN, USA; goat anti-rabbit or anti-mouse IRDye 800 CW secondary antibody from LI-COR Bio Sciences, Lincoln, NE, USA; FastStart Universal SYBR Green Master and LightCycler96 from Roche, Nutley, NJ, USA; and BCA protein Assay kit and protein RIPA lysis buffer from Beyotime Institute of Biotechnology, Haimen, JS, China.

### Animals

The CBS heterozygous knockout C57BL/6 mice (CBS^+/−^) were obtained from Professor Rui Wang of Lakehead University (Thunder Bay, ON, Canada). The CBS^−/−^, CBS^+/−^, and CBS^+/+^ (wild-type) mice were verified by RT-PCR^6^. The mice were housed under specific pathogen-free conditions at 22 ± 2 °C with a relative humidity of 60–65% and maintained under a 12-h light/12-h dark cycle with ad libitum access to food and water as previously described^38^. All animal care and experimental protocols were performed according to the Animal Management Rules of the Ministry of Health of China, and approved by the Animal Ethics Committees of Fudan University (NDFC31271132) and The Chinese University of Hong Kong (GRF14111815).

### Sampling of blood and tissues

Animals were anesthetized with 1% pentobarbital sodium (40 mg/kg body weight, intraperitoneally) and decapitated. Blood samples were collected into syringes containing EDTA-2K for the determination of RBC, Hb, HCT,MCH, MCV as described previously^39^, dropped on the slide, and fixed with methanol and stained using Wright-Giemsa Stain after being pushed forward to the other end of the slide. Mice were perfused with phosphate-buffered saline (PBS), and the liver, spleen and kidney were removed, rinsed in PBS, dried, weighed, and stored in a freezing chamber at −70 °C for subsequent measurements^40,41^. The femur was harvested, cut on one side, and bone marrow was pressed on the side of the slide and mixed with a small amount of fetal bovine serum according to Lozier andCalvo^42^.

### Bone marrow-derived macrophages

BMDMs were prepared as described^43^ with some modifications. In brief, the bone was cut from the knee joint. Roswell Park Memorial Institute medium from a 10-mL syringe with a 26-G needle was flushed into the joint and then collected. The entire medium was incubated with NH4Cl solution on ice for 10 min to remove red blood cells, which were then collected for centrifuging at 1000 rmp at 4 °C for 5 min. Then, BMDMs were washed, cultured in differentiation medium (Roswell Park Memorial Institute medium with 40 ng/mL macrophage colony-stimulating factor), and incubated in fresh medium every 2–3 days.

### Western blot analysis

The tissues were washed and homogenized by protein RIPA lysis buffer as described previously^44,45^. Soluble proteins were collected after centrifugation at 13200 rpm for 15 min at 4 °C and protein content was determined using the BCA protein assay reagent kit. Aliquots of the extract containing 40 μg of protein were loaded and run on a single track of 10% SDS–PAGE and then transferred onto a pure nitrocellulose membrane (Bio-Rad). The blots were blocked with 5% non-fat milk and then incubated overnight at 4^o^C with primary antibodies: rabbit anti-CBS (1:1,000), mouse anti-TfR1 (1:500), rabbit anti-DMT1 (1:1,000), rabbit anti-Fpn1 (1:1,000), rabbit anti-Ft-L (1:1,000), rabbit anti-Ft-H (1:1,000), rabbit anti-EPO (1:100), and rabbit anti-HIF-2α (1:500). After being washed three times, the blots were incubated with goat anti-rabbit (1:1000) or anti-mouse IRDye800 CW secondary antibody (1:5000) for 2 h at room temperature. The intensities of the specific bands were detected and analyzed by the Odyssey infrared imaging system (Li-Cor, Lincoln, NE, USA). Anti-β-actin (1:2,000) was used as internal protein controls.

### Isolation of total RNA and quantitative real-time PCR

The extraction of total RNA and preparation of cDNA were performed using TRIzol reagent and the AevertAid FirstStrand cDNA Synthesis Kit respectively, in accordance with the instructions of the manufacturers. Real-time PCR was carried out by RT-PCR instrument (LC96, Roche, Switzerland) using Fast Start Universal SYBR Green Master and the Light Cycler96. The specific pairs of primers of mouse TfR1, DMT1, Fpn1, Ft-L, IRP1 (iron regulatory proteins 1), IRP2 (iron regulatory proteins 2), EPO, EPOR, ALAS2, FECH, FLVCR, ERFE, and β-actin, are listed in Supplementary Table 1. The cycle threshold value of each target gene was normalized to that of the β-actin mRNA^46,47^. Relative gene expression was calculated by the 2-ΔΔCt method.

### Immunofluorescence staining

Immunofluorescence staining was carried out as described^48,49^. In brief, femurs decalcified in 10% EDTA and all tissues were immersed in cryo-embedding medium and then sectioned into 10-mm thick slices using a cryotome (Leica Microsystems, Wetzlar, Germany). Slices were blocked with 3% bovine serum albumin for 2 h and incubated overnight at 4 °C with primary antibodies: rabbit anti-HIF-2α (1:50) and rabbit anti-EPO (1:10). After being washed with 0.01 M PBS three times, the slides were incubated with Alex Fluor 488 or 594-conjugated secondary antibodies for 1 h at room temperature and then with Hoechest 33342 (5 mg/ml) for 15 min at 37 °C. Negative controls received an identical treatment except for the primary antibody and showed no positive signal.

### Immunohistochemistry staining

Hydrogen peroxide was used to quench endogenous peroxidase activity^50^. Sections were immunohistochemically stained with mouse anti-TfR1 (1:50), rabbit anti-DMT1 (1:50), rabbit anti-Fpn1 (1:50) and rabbit anti-Ft-L (1:100), rabbit anti-Ft-H (1:100), anti-p-JAK2 (1:100), and counterstained with hematoxylin. Signal was detected using a DAB substrate following the manufacturer’s recommendations. The immunostaining images were scanned randomly under a confocal fluorescence microscope and light microscope (Olympus, Tokyo, Japan) by a single investigator who was blind to sample identity.

### Prussian blue staining

Prussian blue staining was performed using freshly prepared 5% potassium hexacya-noferrate and 5 % hydrochloric acid^51^. Sections were rinsed in water and counterstained with nuclear fast red, dehydrated, and covered. The images were observed under a light microscope (Olympus, Tokyo, Japan).

### Statistical analysis

Statistical analysis was performed using one-way analysis of variance and Tukey’s method was used for multiple pair-wise comparisons. All data are expressed as the mean ± SEM. A probability value of *P* < 0.05 was taken to be statistically significant.

## Supplementary information

Supp-Tables 4-5 X Supp-Fig1

Supp-Tables 1-3
