# Supplementary material for: Cystathionine β-synthase (CBS) deficiency suppresses erythropoiesis by disrupting expression of heme biosynthetic enzymes and transporter
Source: Cell Death Dis. 2019 Sep 24;10(10):708. doi: 10.1038/s41419-019-1951-0 (PMC6760157; doi:10.1038/s41419-019-1951-0)
Supplement: Supplementary file 1 — Supp-Tables 4-5 X Supp-Fig1 [file 41419_2019_1951_MOESM1_ESM.doc]

**Supplement Table 4. Classification counts of** 100 erythroblasts and computes percentages of each type of sideroblast in bone marrow smear with Prussian blue stain.

| Genotype | Types of sideroblast (%) | | | | | Sideroblast  (%) | Total |
| --- | --- | --- | --- | --- | --- | --- | --- |
|  | I | II | III | IV | 0 |  |  |
| CBS+/+ | 7 | 16 | 34 | 2 | 41 | 59 | 100 |
|  | 4 | 13 | 19 | 0 | 64 | 36 | 100 |
|  | 3 | 15 | 23 | 0 | 59 | 41 | 100 |
| CBS+/- | 5 | 18 | 33 | 0 | 44 | 56 | 100 |
|  | 4 | 17 | 28 | 1 | 50 | 50 | 100 |
|  | 3 | 20 | 27 | 2 | 48 | 52 | 100 |
|  | 3 | 13 | 24 | 0 | 60 | 40 | 100 |
|  | 3 | 14 | 27 | 3 | 53 | 47 | 100 |
| CBS-/- | 6 | 18 | 27 | 28 | 21 | 79 | 100 |
|  | 3 | 16 | 33 | 12 | 36 | 64 | 100 |
|  | 5 | 14 | 27 | 15 | 39 | 61 | 100 |

**Supplement Table 5.** CBS deficiency increased the number of sideroblasts and iron content in erythroblasts of bone marrow

|  |  | CBS+/+ | CBS+/- | CBS-/- |
| --- | --- | --- | --- | --- |
| Types of sideroblast (%) | I | 4.7 ± 1.20 | 3.6 ± 0.40 | 4.7 ± 0.88 |
|  | II | 14.7 ± 0.88 | 16.4± 1.29 | 16.0± 1.16 |
|  | III | 25.3 ± 4.49 | 27.8 ± 1.46 | 29.0 ± 2.00 |
|  | IV | 0.67 ± 0.67 | 1.20 ± 0.58 | 18.3 ± 4.91**^##^ |
|  | 0 | 54.7 ± 6.98 | 51.0 ± 2.68 | 32.0 ± 5.57* ^#^ |
| Sideroblast (%) | | 45.3 ± 6.98 | 49.0 ± 2.68 | 68.0 ± 5.57* ^#^ |
| Total (%) | | 100 | 100 | 100 |

Data are expressed as means ± SEM. * P < 0.05, ** P < 0.01 versus CBS+/+. ^#^ P < 0.05, ^##^ P < 0.01 versus CBS+/-.

**a**

**b**

**c**


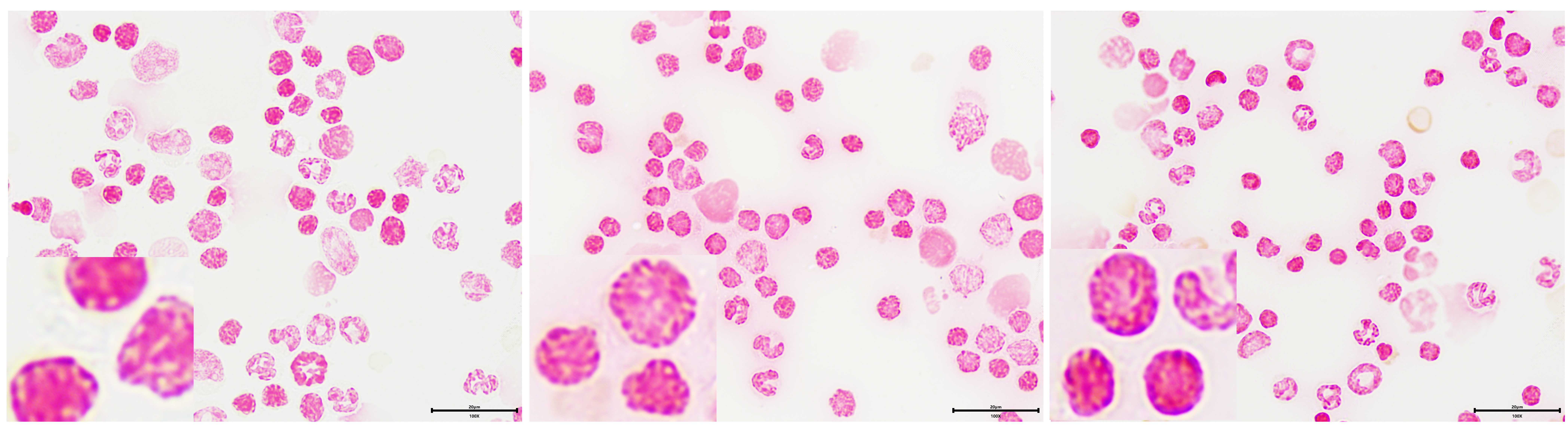


**20µm**

**Supplement Fig.1 Sideroblasts in erythroblasts of bone marrow smear with Prussian blue stain. a CBS+/+, b CBS+/-, c CBS-/-.**

**Method**

**Erythroblasts iron status in bone marrow**

Bone marrow was collected from the femur. Smears were prepared from marrow fragments, colored with Prussian blue stain, and interpreted by a senior hematologist who was blind to sample identity (Stancu et al. 2010). Under oil microscope, 100 erythroblasts were classification counted, and erythroblasts with blue particles on Prussian blue stain were defined as sideroblasts. According to the number of siderotic granules, sideroblasts were classifified as type 0 (0 granules), type I (1-2 granules), type II (3-5 granules), or type III ( 6-10 granules), type IV (more than 10 granules). Percentages of each type of sideroblast were computed.

**Result**

**CBS deficiency increased the number of sideroblasts and iron content in erythroblasts of bone marrow**

Microscopic examination of a Prussian blue stained bone marrow smears is widely regarded as the "gold standard" for the assessment of the iron status in erythroblasts (Bableshwar et al. 2013; Phiri et al. 2009). We examined the changes of iron status in bone marrow erythroblasts induced by CBS deficiency. We found that the number of sideroblasts was significantly increased in bone marrow of CBS -/- mice as compared with CBS +/- and CBS +/+ mice (Supplement Table 4 & 5). The percentage of type IV sideroblast was remarkable higher in CBS-/- mice (Supplement Fig 1. c) than those in CBS +/- (Supplement Fig 1. b) and CBS +/+ mice (Supplement Fig 1. a). These findings demonstrated that CBS deficiency could induce a significant increase in the number of sideroblasts and the iron content in erythroblasts of bone marrow.

**References:**

[Bableshwar RS](https://www.ncbi.nlm.nih.gov/pubmed/?term=Bableshwar RS[Author]&cauthor=true&cauthor_uid=23924552), [Roy M](https://www.ncbi.nlm.nih.gov/pubmed/?term=Roy M[Author]&cauthor=true&cauthor_uid=23924552), [Bali A](https://www.ncbi.nlm.nih.gov/pubmed/?term=Bali A[Author]&cauthor=true&cauthor_uid=23924552), [Patil PV](https://www.ncbi.nlm.nih.gov/pubmed/?term=Patil PV[Author]&cauthor=true&cauthor_uid=23924552), [Inumella S](https://www.ncbi.nlm.nih.gov/pubmed/?term=Inumella S[Author]&cauthor=true&cauthor_uid=23924552). Intensive method of assessment and classification of the bone marrow iron status: a study of 80 patients. [Indian J Pathol Microbiol.](https://www.ncbi.nlm.nih.gov/pubmed/?term=Intensive+method+of+assessment+and+classification+of+the+bone+marrow+iron+status:+A+study+of+80+patients) 2013;56(1):16-9. doi: 10.4103/0377-4929.116142.

[Phiri KS](https://www.ncbi.nlm.nih.gov/pubmed/?term=Phiri KS[Author]&cauthor=true&cauthor_uid=19638538), [Calis JC](https://www.ncbi.nlm.nih.gov/pubmed/?term=Calis JC[Author]&cauthor=true&cauthor_uid=19638538), [Kachala D](https://www.ncbi.nlm.nih.gov/pubmed/?term=Kachala D[Author]&cauthor=true&cauthor_uid=19638538), [Borgstein E](https://www.ncbi.nlm.nih.gov/pubmed/?term=Borgstein E[Author]&cauthor=true&cauthor_uid=19638538), [Waluza J](https://www.ncbi.nlm.nih.gov/pubmed/?term=Waluza J[Author]&cauthor=true&cauthor_uid=19638538), [Bates I](https://www.ncbi.nlm.nih.gov/pubmed/?term=Bates I[Author]&cauthor=true&cauthor_uid=19638538), [Brabin B](https://www.ncbi.nlm.nih.gov/pubmed/?term=Brabin B[Author]&cauthor=true&cauthor_uid=19638538), [van Hensbroek MB](https://www.ncbi.nlm.nih.gov/pubmed/?term=van Hensbroek MB[Author]&cauthor=true&cauthor_uid=19638538). Improved method for assessing iron stores  in the bone  marrow. [J Clin Pathol.](https://www.ncbi.nlm.nih.gov/pubmed/?term=Improved+method+for+assessing+iron+stores+in+the+bone+marrow) 2009;62(8):685-9. doi: 10.1136/jcp.2009.064451.

[Stancu S](https://www.ncbi.nlm.nih.gov/pubmed/?term=Stancu S[Author]&cauthor=true&cauthor_uid=20079959), [Stanciu A](https://www.ncbi.nlm.nih.gov/pubmed/?term=Stanciu A[Author]&cauthor=true&cauthor_uid=20079959), [Zugravu A](https://www.ncbi.nlm.nih.gov/pubmed/?term=Zugravu A[Author]&cauthor=true&cauthor_uid=20079959), [Bârsan L](https://www.ncbi.nlm.nih.gov/pubmed/?term=Bârsan L[Author]&cauthor=true&cauthor_uid=20079959), [Dumitru D](https://www.ncbi.nlm.nih.gov/pubmed/?term=Dumitru D[Author]&cauthor=true&cauthor_uid=20079959), [Lipan M](https://www.ncbi.nlm.nih.gov/pubmed/?term=Lipan M[Author]&cauthor=true&cauthor_uid=20079959), [Mircescu G](https://www.ncbi.nlm.nih.gov/pubmed/?term=Mircescu G[Author]&cauthor=true&cauthor_uid=20079959). Bone marrow iron, iron indices, and the response to intravenous iron in patients with non-dialysis-dependent CKD. [Am J Kidney Dis.](https://www.ncbi.nlm.nih.gov/pubmed/20079959) 2010;55(4):639-47. doi: 10.1053/j.ajkd.2009.10.043.
