# Supplementary material for: Cystathionine β-synthase (CBS) deficiency suppresses erythropoiesis by disrupting expression of heme biosynthetic enzymes and transporter
Source: Cell Death Dis. 2019 Sep 24;10(10):708. doi: 10.1038/s41419-019-1951-0 (PMC6760157; doi:10.1038/s41419-019-1951-0)
Supplement: Supplementary file 2 — Supp-Tables 1-3 [file 41419_2019_1951_MOESM2_ESM.docx]

**Supplement - Table 1.**

**The speciﬁc pairs of primers**

|  | **Forward（5′-3′）** | **Reverse（5′-3′）** |
| --- | --- | --- |
| β-actin | AAATCGTGCGTGACATCAAAGA | GCCATCTCCTGCTCGAA GTC |
| DMT1(Slc11a2) | GCGGCCAGTGATGAGTGAGT | ATGCCACCGGCAATCCT |
| EPO | TCCCCCACGCCTCATCT | TTTCTGCCTCCTTGGCCTCTA |
| EPOR | TCATACCAGCTCGAGGGTGA | GGTGATAGCGAGGAGAACCG |
| FPN1(Slc40a1) | TCACCTGGCTACGTCGAAAAT | GCTGGGCTAGTCCTGAGAATAGAC |
| FTL | CGGGCCTCCTACACCTACCT | CCCTCCAGAGCCACGTCAT |
| IRP1 | ACAGGCCGCGAGGAAGA | GAAACATGCCTACAGCCTGAAGAT |
| IRP2 | GCCATAGCAGGCACAGTGAATA | TTTCCTTGCCCGTAGAGTCAGT |
| Nrf2 | CGAGATATACGCAGGAGAGGTAAGA | GCTCGACAATGTTCTCCAGCTT |
| TfR1 | CTCAGTTTCCGCCATCTCAGT | GCAGCTCTTGAGATTGTTTGCA |

**Supplement - Table 2.**

**CBS deﬁciency suppresses erythropoiesis**

**and increases the ratio of granulocyte / erythrocyte in bone marrow**

|  | **CBS+/+** | **CBS+/-** | **CBS-/-** |
| --- | --- | --- | --- |
| Erythroid Cells total | 194.3±2.186 | 178±9.539 | 130.7±4.807*****^##^** |
| Prorubricyte | 55.67±5.239 | 46.67±5.364 | 46.33±9.871 |
| [Polychromatic](javascript:;) [erytehroblast](javascript:;) | 14.33±3.930 | 16.33±3.180 | 12.33±5.783 |
| [Metarubricyte](javascript:;) | 124.3±7.860 | 114.7±6.936 | 72.00±10.410***^#^** |
| Ratio of granulocyte/ erythrocyte | 0.98±0.09815 | 1.17±0.1058 | 2.09±0.1937****^##^** |

Data are expressed as means ± SEM. **p* < 0.05, ***p* < 0.01, ****p* < 0.001 versus CBS+/+. ^#^*p* < 0.05, ^##^*p* < 0.01 versus CBS+/-.

**Supplement - Table 3.**

**Classified count of 500 cells except megakaryocytes in bone marrow smear**

|  | **CBS+/+** | | | **CBS+/-** | | | **CBS-/-** | | |
| --- | --- | --- | --- | --- | --- | --- | --- | --- | --- |
| Lymphocyte and Other cells | 109 | 91 | 146 | 117 | 125 | 104 | 122 | 96 | 78 |
| Erythroid Cells total | 197 | 190 | 196 | 194 | 161 | 179 | 140 | 124 | 128 |
| *Pronormoblast* | 0 | 0 | 0 | 0 | 0 | 1 | 0 | 0 | 0 |
| *Prorubricyte* | 66 | 52 | 49 | 57 | 39 | 44 | 66 | 35 | 38 |
| [*Polychromatic*](javascript:;)*[erytehroblast](javascript:;)* | 22 | 9 | 12 | 10 | 19 | 20 | 22 | 2 | 13 |
| *[Metarubricyte](javascript:;)* | 109 | 129 | 135 | 127 | 103 | 114 | 52 | 87 | 77 |
| [Granulocyte](javascript:;) | 194 | 219 | 158 | 189 | 214 | 217 | 238 | 280 | 294 |
| Ratio of granulocyte/erythrocyte | 0.98:1 | 1.15:1 | 0.81:1 | 0.97:1 | 1.33:1 | 1.21:1 | 1.70:1 | 2.26:1 | 2.30:1 |
| Total number of cells counted | 500 | 500 | 500 | 500 | 500 | 500 | 500 | 500 | 500 |
